# Urolithin A alleviates vascular remodeling through mitochondrial SIRT3-mediated SOD2 deacetylation and antioxidation in hypertensive rats

**DOI:** 10.1080/13510002.2026.2622255

**Published:** 2026-02-06

**Authors:** Min Dai, Yi-Ming Wang, Hong-Ke Dong, Xiao-Yu Xu, Jing-Xiao Wang, Guo-Qing Zhu, Fen Zheng

**Affiliations:** a Key Laboratory of Targeted Intervention of Cardiovascular Disease, Collaborative Innovation Center for Cardiovascular Disease Translational Medicine, and Department of Physiology, Nanjing Medical University, Nanjing, People’s Republic of China; b Taizhou School of Clinical Medicine, The Affiliated Taizhou People's Hospital of Nanjing Medical University, Taizhou, People’s Republic of China

**Keywords:** Urolithin A, mitochondrial reactive oxygen species, superoxide dismutase 2, hypertension, vascular remodeling, vascular smooth muscle cell

## Abstract

**Objectives:**

Urolithin A (UA) is a natural polyphenolic compound produced by gut bacteria. Vascular remodeling contributes to hypertension, and vascular smooth muscle cells (VSMCs) proliferation and migration are important processes in vascular remodeling.

**Methods:**

VSMCs were obtained from the thoracic aorta of Wistar-Kyoto rats (WKY) and spontaneously hypertensive rats (SHR). Intraperitoneal injections of UA (50 mg/kg, every 2 days for 4 weeks) were performed in SHR.

**Results:**

UA attenuated proliferation and migration, reduced mitochondrial reactive oxygen species (mitoROS) levels, and increased SOD2 activity in VSMCs of SHR, which were prevented by SOD2 knockdown. UA promoted mitochondrial short-length SIRT3 (SL-SIRT3) production and SOD2 deacetylation. SIRT3 inhibitor 3-TYP abolished the effects of UA on SOD2 deacetylation, mitoROS levels and VSMCs proliferation and migration. Repeated intraperitoneal injection of UA every 2 days for 4 weeks attenuated vascular remodeling and hypertension, increased SL-SIRT3 levels and SOD2 activity, and reduced SOD2 acetylation and mitoROS levels in aorta and mesenteric arteries of SHR.

**Conclusion:**

UA attenuates VSMCs proliferation and migration in SHR by increasing mitochondrial SL-SIRT3 level, and subsequent SOD2 deacetylation and mitoROS reduction in SHR. Long-term administration of UA attenuates vascular remodeling, hypertension and oxidative stress in SHR.

## Introduction

Urolithin A (UA, C_13_H_8_O_4_) is a natural polyphenolic compound containing an α-benzo-coumarin scaffold, which is produced by gut bacteria [1]. The bacteria in the colon convert ingested polyphenol compounds, including ellagitannins and ellagic acid, to UA in animals and humans [2]. Ellagitannins and ellagic acid are abundant in a variety of foods such as pomegranate, nuts and berries [3]. UA promotes mitophagy and improves mitochondrial function [4], and exhibits anti-tumor, anti-inflammatory, antioxidant, and anti-aging properties [5]. Some preclinical studies show that UA protects against aging, and might be a potential therapeutic agent for several diseases [3–5].

Vascular remodeling contributes to the development of hypertension, related complications and end-organ damage [6]. Intervention of vascular remodeling may be an important therapeutic strategy for preventing the deterioration of hypertension and the occurrence of cardiovascular events [7–9]. Vascular smooth muscle cells (VSMCs) are the main component of the arterial walls. The VSMCs proliferation and migration are important pathological processes in vascular remodeling [10].

Reactive oxygen species (ROS) serve as signaling molecules for a variety of biological functions in the physiological state. Excessive ROS due to the imbalance between the ROS production and the antioxidant processes leads to oxidative stress and subsequent pathological changes [11]. Mitochondria and NADPH oxidases (NOX) are the two major sources of ROS production [12]. The mitochondria are a source of mitochondrial ROS (mitoROS) including superoxide anion (O_2_^−^), hydrogen peroxide (H_2_O_2_) and hydroxyl radical [13]. The mitoROS can leak into the cytoplasm and modify cellular activity [14]. The precise regulation of ROS production and clearance is important for cellular homeostasis. The O_2_^−^ is converted to H_2_O_2_ through SOD1 (CuZn-SOD) in cytoplasm, SOD2 (Mn-SOD) in mitochondria, and SOD3 (EC-SOD) in extracellular space, and the H_2_O_2_ is then converted to water by catalase or peroxidase systems [15]. The excessive mitoROS production leads to abnormal phenotypic changes of VSMCs [16], and NOX-derived ROS is involved in vascular remodeling [17]. Kidney is innervated by renal nerves [18], and selective removal of renal afferent fibers attenuates vascular remodeling and oxidative stress in spontaneously hypertensive rats (SHR) [19]. Oxidative stress contributes to vascular remodeling in hypertension [20–22]. It has been shown that UA attenuates H_2_O_2_-induced oxidative stress in the Neuro-2a cells [23] and the SK-N-MC cells [24], and ischemia-reperfusion-induced oxidative stress in the myocardium [25]. However, it is unknown whether UA may alleviate vascular remodeling and oxidative stress in hypertension. Furthermore, the exact mechanism of UA in antioxidation remains unclear. This study aimed to reveal the roles and underlying mechanisms of UA in vascular remodeling and oxidative stress in SHR.

## Materials and methods

### Animals

Total 12 male Wistar-Kyoto rats (WKY) and 24 male SHR (Vital River, Beijing, China) aged at 8 weeks were used in the present study. SHR is a genetic model bred from progenitor WKY, therefore WKY was used as the control. Experiments were approved by the Experimental Animal Care and Use Committee of Nanjing Medical University (No. IACUC-2308018). Rats were housed in a temperature- and humidity-controlled house with 12 h/12 h light/dark cycle. Standard laboratory chow and tap water were freely available. All rats meet the criterion that systolic blood pressure < 140 mm Hg for WKY, and >160 mm Hg for SHR. The rat was euthanized by intravenous injection of pentobarbital sodium (150 mg/kg).

### Culture of VSMCs

WKY and SHR were anesthetized with pentobarbital sodium (50 mg/kg, i.p.). The thoracic aorta was carefully isolated and stripped of adherent perivascular tissue. The aorta was cut open, and the media was obtained by peeling off the intima. The media was treated with DMEM containing 0.4% collagenase for 30 min. After centrifuging at 300×g for 5 min at room temperature, the cells were collected and cultured in DMEM containing 10% fetal bovine serum (FBS). Primary VSMCs between the third and the fifth passages were used in the study [26].

### Evaluation of cell proliferation and migration

EdU incorporation assay kit (Cat. #C0078S) and CCK-8 kit (Cat. #C0041) were obtained from Beyotime (Shanghai, China) to evaluate VSMCs proliferation [27]. VSMCs migration was evaluated with the Boyden chamber assay and wound healing assay [28]. For EdU incorporation assay, Hoechst33342 was used to label the nuclei (blue color), and the percentage of EdU-positive cells (red color) was calculated for EdU incorporation assay. For the measurements with CCK-8 kit, absorbance was measured at 450 nm with a microplate reader. For the Boyden chamber assay, the cells that moved to the lower surface of the membrane were stained with 1% crystal violet. The average number of stained cells in five randomly selected fields was counted. For the wound healing assay, the wound healing was photographed at 0 and 24 h, and the migrated distance was measured and calculated.

### DCF and mitoSOX fluorescence analyses

Total ROS levels and mitochondrial superoxide levels were respectively evaluated with mitoSOX fluorescence analyses as previously described [29]. Briefly, VSMCs were stained with DCFH-DA (10 μM, Cat. #S0033S, Beyotime, Shanghai, China) in the dark for 30 min before being washed with PBS for DCF fluorescence analyses, or incubated with mitoSOX (5 μM) at 37°C for 10 min for mitoSOX fluorescence analyses (510 nm excitation and 580 nm emission).

### Lucigenin chemiluminescence assay

Superoxide level and NOX activity were measured with lucigenin chemiluminescence assay [21,30]. Photon emission was initiated by adding dark-adapted lucigenin (5 μM) for measuring superoxide level, or by adding both dark-adapted lucigenin (5 μM) and NADPH (100 μM) for measuring NOX activity. Average values were obtained by measuring 10 times within 10 min, and a buffer containing lucigenin (5 µM) was measured as background chemiluminescence. The values were expressed in mean light unit/min/mg protein.

### SOD2 knockdown

Small-interfering RNA (siRNA) for SOD2 knockdown and negative control siRNA (NC) were commercially obtained from General Bio (Chuzhou, Anhui, China). The siRNA sequences were 5′-GAGAGUUGCUGGAGGCUAUTT-3′ (Forward) and 5′-AUAGCCUCCAGCAACUCUCTT-3′ (Reverse). VSMCs were seeded on 6-well plates, and transfected with NC or SOD2-siRNA (50 nM) using Lipofect5000 transfection reagent (Cat. #21023, BIOG, Changzhou, Jiangsu, China) following manufacturer’s instructions for 48 h. The transfection efficiency was identified by SOD2 expression in VSMCs.

### Measurement of SOD2 activity

SOD2 activity in VSMCs was examined with the Hydroxylamine Method using a CuZn/Mn Superoxide Dismutase (CuZn-SOD/Mn-SOD) Activity Assay Kit (Cat. #E-BC-K022-M, Elabscience, Wuhan, Hubei, China) following the manufacturer’s instructions. Absorption was measured at 550 nm using a Microplate Reader (BioTek Instruments, Winooski, VT, USA).

### Measurement of H_2_O_2_ level

Hydrogen peroxide assay kit (Cat. #S0038, Beyotime, Shanghai, China) was used for H_2_O_2_ quantification in VSMCs according to the manufacturer’s instructions. Absorbance was measured at 560 nm. Hydrogen peroxide (H₂O₂) levels were expressed as nmol/mg protein.

### Molecular docking analysis

The structure of SIRT3 protein (ID: 3GLS) and UA (ID: 5488186) were obtained from RCSB Protein Data Bank database (https://www.rcsb.org/) and PubChem database (https://pubchem.ncbi.nlm.nih.gov/), respectively. The 3D format of UA was made with Open Babel software (Version 2.3.2, https://openbabel.org) [31]. The 3D molecular docking model was made using AutoDock software (Version 4.2.4, https://autodock.scripps.edu/) and PyMOL software (Version 3.1, https://pymol.org/) [32]. The 2D molecular docking model was produced with LigPlot + software (Version 2.2, https://www.ebi.ac.uk/thornton-srv/software/LigPlus/) [33].

### Cellular thermal shift assay (CETSA)

CETSA was used to examine the ligand binding in living cells [34]. VSMC lysates were separated into two parts and incubated with 1% DMSO and UA (25 μM) for an hour. Then cell lysates were heated between 40 and 61°C, respectively, for 3 min and cooled at 25°C for another 3 min. The supernatants were collected, and SIRT3 and GAPDH proteins were detected by Western blotting.

### Surface plasmon resonance (SPR)

SPR was used to identify the binding affinities of UA with SIRT3 with a Biacore™ X100 SPR system (Cytiva, Uppsala, Sweden). SIRT3 was diluted to 40 μg/mL in sodium acetate (pH 4.0) fixation buffer. The recombinant protein was immobilized onto a CM5 chip (Cat:#29149603, Cytiva, Chicago, IL, USA) through EDC/NHS coupling. UA was injected across a concentration gradient (from 100 to 3.13 μM) at a constant flow rate of 10 μL/min. The binding and dissociation phases were set to 90 and 120 s, respectively. All SPR data processing and analyses were performed using BiaEvaluation Software 3.0 (Cytiva, Chicago, IL, USA). All binding resonance signals were presented after double-reference (blank surface reference and blank buffer reference) correction. The steady-state binding curve was fitted using the Kinetics binding mode to obtain the Kd value [35,36].

### Separation of proteins of subcellular components

Mitochondrial Isolation and Protein Extraction Kit (Cat. #PK10016, Proteintech Group, Inc., Chicago, IL, USA) was used to extract mitochondria according to the manufacturer's instructions. Briefly, cells were collected in cold PBS and centrifuged at 500×g for 5 min at 4°C. After removing the supernatant, the sedimentation was suspended in 1 ml buffer A containing 1% phenylmethanesulfonyl fluoride (PMSF) and then homogenized on ice with a glass homogenizer. The homogenates were added to the same volume of buffer B and centrifuged at 600×g for 10 min, and then the supernatant was centrifuged at 10,000×g for 10 min at 4°C. The supernatant constituted the non-mitochondrial cellular fraction (mitochondria-removed cellular components). The pellet was mitochondria, and was suspended in an appropriate volume of mitochondrial lysis buffer containing 1% PMSF. After incubation on ice for 30 min, the sample was centrifuged at 10,000×g for 5 min at 4 °C. The supernatant was collected as the mitochondrial protein extract.

Nuclear/cytosol fractionation kit (Cat. #P0027, Beyotime, Shanghai, China) was used to extract nuclear proteins according to the manufacturer’s instructions. Simply, VSMCs in a six-well plate were rinsed twice in cold PBS. The cells were collected and suspended in Reagent A containing PMSF at a final concentration of 1 mM with vigorous vortex for 5 s. After incubation on ice for 10 min, Reagent B was added, and then incubated for 1 min on ice. The sample was centrifuged at 16,000×g for 5 min at 4°C, and the supernatant containing cytoplasmic protein was removed. The precipitates were lysed in Nuclear Protein Isolation Reagent containing PMSF at a final concentration of 1 mM for 30 min. After centrifugation at 16,000×g for 5 min at 4°C, the supernatant containing nuclear protein was harvested.

### Western blot analysis

VSMCs or arteries were homogenized in lysis buffer. Proteins in samples were separated with 10% SDS-PAGE and transferred to a PVDF membrane. The membrane was incubated with the first antibody overnight at 4°C, and then with the secondary antibody conjugated with HRP at room temperature for 1 h.

### RT–PCR

SIRT3 mRNA levels in VSMCs of WKY and SHR were examined with quantitative real-time PCR analysis. GAPDH was used as an internal control. Relative values were obtained with ΔΔCt method. The sequence of primers was obtained through the Basic Local Alignment Search Tool (BLAST, https://blast.ncbi.nlm.nih.gov/Blast.cgi). The sequence and accession code were listed as follows. SIRT3: Forward, 5′-TTCTGCGGCTCTACACACAG-3′; Reverse, 5′-ACGTCAGCCCGTATGTCTTC-3′ (code: FQ218084.1). GAPDH: Forward, 5′-TTCCAGGAGCGAGATCCCGCTAAC-3′; Reverse, 5′-TTCAGGTGAGCCCCAGCCTTCT -3′ (code: FQ214999.1).

### Blood pressure measurements

Systolic blood pressure (SBP) and heart rate (HR) in the waking state were obtained from the tail artery of rats with a noninvasive computerized tail-cuff system (NIBP, ADInstruments, Sydney, New South Wales, Australia) once a week. Before the measurements, the rats were warmed for 15 min at 28°C to allow a better detection of tail artery pulsations. The SBP and HR values were obtained through averaging the values of 10 measurements. Mean arterial pressure (MAP) and HR under anesthesia were measured through direct left common carotid artery intubation with a polyethylene catheter (PE50) filled with heparinized saline. HR was obtained according to the arterial pressure waves.

### Masson’s staining

The aorta and mesentery artery (MA) of WKY and SHR were prefixed. Paraffin-embedded sections were stained with Masson’s trichrome staining. The images of sections were obtained with a light microscope and analyzed with Image Pro Plus 6.0 (Media Cybernetics, Rockville, MD, USA). Media thickness, lumen diameter, and their ratios were used to evaluate the vascular remodeling.

### Chemicals and antibodies

UA (Cat. #HY-100599), 3-TYP (Cat. #HY-108331), CsA (Cat. #HY-B0579) and mitoSOX (Cat. #HY-D1055) were obtained from Med Chem Express (Monmouth Junction, NJ, USA). SIRT3 (Cat: #S37-30EG) was bought from Sino Biological Inc. (Beijing, China). Y040-7904 was commercially provided by ChemDiV Inc. (San Diego, CA, USA). Antibodies against NOX1 (Cat. #17772-1-AP), NOX2 (Cat. #19013-1-AP), NOX4 (Cat. #14347-1-AP), SOD2 (Cat. #24127-1-AP), COXIV (Cat. #11242-1-AP), PCNA (Cat. #10205-2-AP) and β-actin (Cat. #66009-1-Ig) were bought from Proteintech Group, Inc. (Chicago, IL, USA). Antibodies of p62 (Cat. #ab019012) and SIRT3 (28 kDa, Cat. #ab137037) were obtained from Abcam (Cambridge, MA, USA). Antibodies of SIRT3 (both 44 and 28 kDa, Cat. #FNab07881) were obtained from FineTest (Wuhan, Hubei, China). Antibody of AC-SOD2 (acetyl K68) was bought from HuaBio (Cat. #HA722251, Hangzhou, Zhejiang, China). Antibody against LC3B (Cat. #T55992F) was bought from Abmart (Shanghai, China).

### Statistics and data analysis

Experiments were performed in a random and double-blinded manner. The number for each group is the number of independent experiments. Data were shown in mean ± SD. One-way or two-way ANOVA followed by post-hoc Bonferroni test was used for the comparison among multiple groups.

## Results

### Effects of UA on VSMCs proliferation and migration

**Figure 1.**
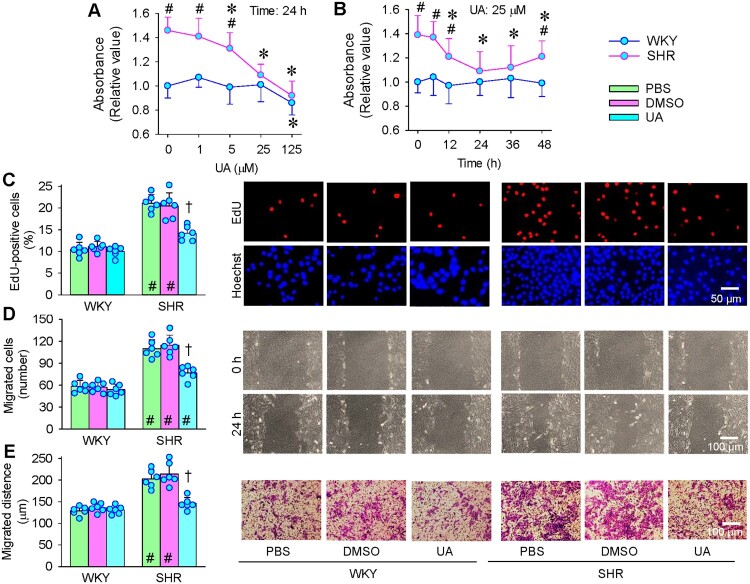
Effects of UA on VSMC proliferation and migration of WKY and SHR. A, dose-effect of UA on VSMC proliferation evaluated with CCK-8 kit. B, time effect of UA on VSMC proliferation evaluated with CCK-8 kit. C, effect of UA (25 μM) on VSMC proliferation evaluated with EdU incorporation assay. D, effect of UA (25 μM) on VSMC migration evaluated with wound healing assay. E, effect of UA (25 μM) on VSMC migration was evaluated with the Boyden chamber assay. Values are mean ± SD. *P < 0.05 vs 0 μM or 0 h; †*P* < 0.05 vs PBS or DMSO; #*P* < 0.05 vs WKY. *n* = 6. Two-way ANOVA followed by Bonferroni post hoc test.

UA inhibited VSMCs proliferation in a dose-dependent manner in SHR, and the significant effects were observed at the doses 5, 25 and 125 μM according to CCK-8 kit method. UA at 5 μM and 25 μM had no significant effects on VSMCs proliferation in WKY, but UA at 125 μM showed significant inhibiting effects (Figure 1(A)). Considering that 125 μM UA may interfere with normal physiological function in WKY or may cause mild cytotoxicity and that 25 μM UA almost normalized the enhanced VSMCs proliferation in SHR, the concentration of 25 μM was used for the following experiments. Based on the time-effects of 25 μM UA, maximal inhibitory effects occurred 24 h after administration of UA in SHR (Figure 1(B)). The inhibitory effects of UA on VSMCs proliferation were further confirmed by EdU incorporation assay (Figure 1(C)). Similarly, UA at 25 μM inhibited VSMCs migration of SHR, but had no significant effects on that of WKY based on the Wound healing assay (Figure 1(D)) and Boyden chamber assay (Figure 1(E)).

### Effects of UA on oxidative stress

**Figure 2.**
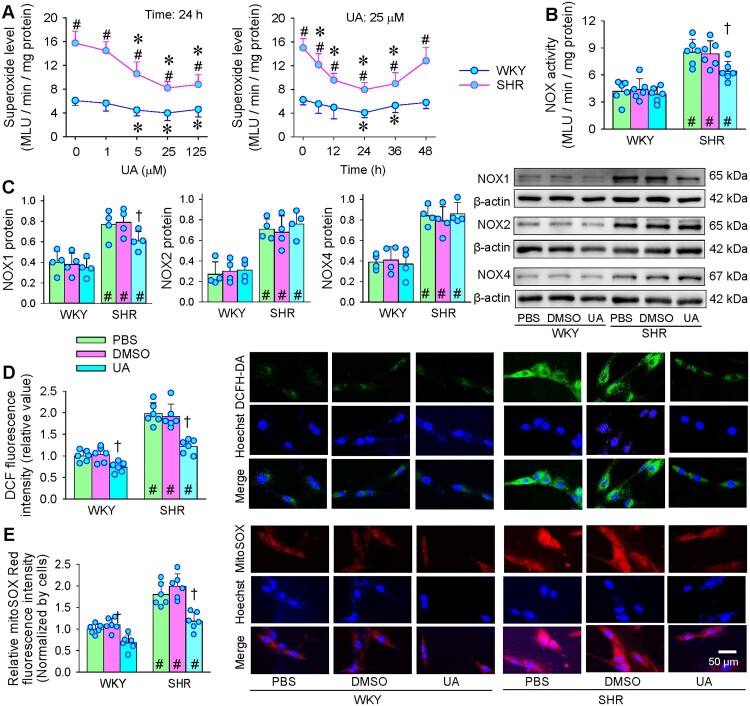
Effects of UA on oxidative stress in VSMCs of WKY and SHR. A, dose-effect and time effect of UA on superoxide production in VSMCs. B, NADPH oxidase (NOX) activity. C, NOX1/2/4 protein expressions. D, relative DCF fluorescence intensity (green) showing intracellular ROS levels. E, relative mitoSOX Red fluorescence intensity (red) showing mitochondrial ROS levels. Nuclei were stained with Hoechst (blue). Values are mean ± SD. *P < 0.05 vs 0 μM or 0 h; †*P* < 0.05 vs PBS or DMSO; #*P* < 0.05 vs WKY. *n* = 6 for A-B & D-E; *n* = 4 for C. Two-way ANOVA followed by Bonferroni post hoc test.

UA at 5, 25 and 125 μM significantly reduced superoxide levels in both WKY and SHR. The maximal effects of UA were observed at the concentration of 25 μM and 24 h after application of UA (Figure 2(A)). UA inhibited NOX activity and NOX1 expression in SHR rather than NOX2 and NOX4 expressions. However, it had no significant effects on NOX activity and NOXs expressions in WKY (Figure 2(B,C)). DCF and mitoSOX fluorescence staining were used to show the total ROS and mitoROS changes. UA reduced total ROS and mitoROS in both strains of rats, and almost normalized the total ROS and mitoROS levels in SHR, suggesting that the roles of UA in inhibiting oxidative stress are mainly attributed to the reduced ROS in mitochondria (Figure 2(D,E)). Furthermore, UA reduced H_2_O_2_ levels in the VSMCs of both WKY and SHR (Figure S1), which may be related to the reduced superoxide levels after application of UA.

### Roles of SOD2 in the effects of UA on oxidative stress, proliferation and migration

**Figure 3.**
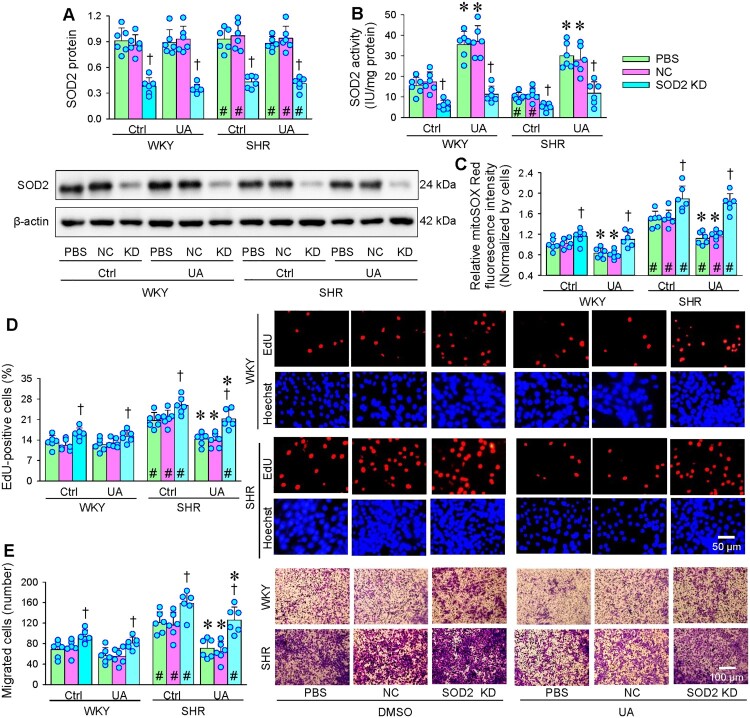
Roles of SOD2 in the effects of UA on oxidative stress, proliferation and migration in VSMCs of WKY and SHR. SOD2 siRNA was used for the knockdown of SOD2 (KD). The cells were treated with PBS, negative control (NC, 50 nmol) or SOD2 siRNA (50 nmol) 48 h before application of UA (25 μM). A, SOD2 protein expression. B, SOD2 activity. C, mitochondrial ROS levels normalized by cell count. D, VSMC proliferation evaluated with EdU incorporation assay. E, VSMC migration was evaluated with the Boyden chamber assay. Values are mean ± SD. *P < 0.05 vs Ctrl; †*P* < 0.05 vs PBS or DMSO; #*P* < 0.05 vs WKY. *n* = 6. Two-way ANOVA followed by Bonferroni post hoc test.

It is known that the antioxidant enzyme SOD2 is mainly located in mitochondria [15], so the roles of SOD2 in the effects of UA were examined in VSMCs of WKY and SHR. UA had no significant effect on SOD2 expression in the VSMCs of both WKY and SHR, and SOD2 knockdown effectively reduced SOD2 expression (Figure 3(A)). Although there was no significant difference in the SOD2 expression in VSMCs between WKY and SHR, the SOD2 activity in VSMCs was lower in SHR than those in WKY, suggesting that the increase of ROS in the VSMCs of SHR may be mainly due to the decrease of SOD2 activity rather than the change of SOD2 expression. UA increased SOD2 activity in VSMCs of both WKY and SHR, and SOD2 knockdown significantly reduced SOD2 activity (Figure 3(B)). MitoROS level in VSMCs was much higher in SHR than in WKY. UA reduced mitoROS levels in VSMCs of both strains, which were reversed by SOD2 knockdown (Figure 3(C)). UA had no significant effect on VSMCs proliferation and migration of WKY, but inhibited the enhanced VSMCs proliferation and migration of SHR. The effects of UA on the VSMCs proliferation and migration in SHR were prevented by the SOD2 knockdown (Figure 3(D,E)). These results suggest that the roles of UA in activating SOD2 attributes to the inhibitory effects of UA on mitoROS production, VSMCs proliferation, and migration.

### UA and SIRT3 molecular docking

**Figure 4.**
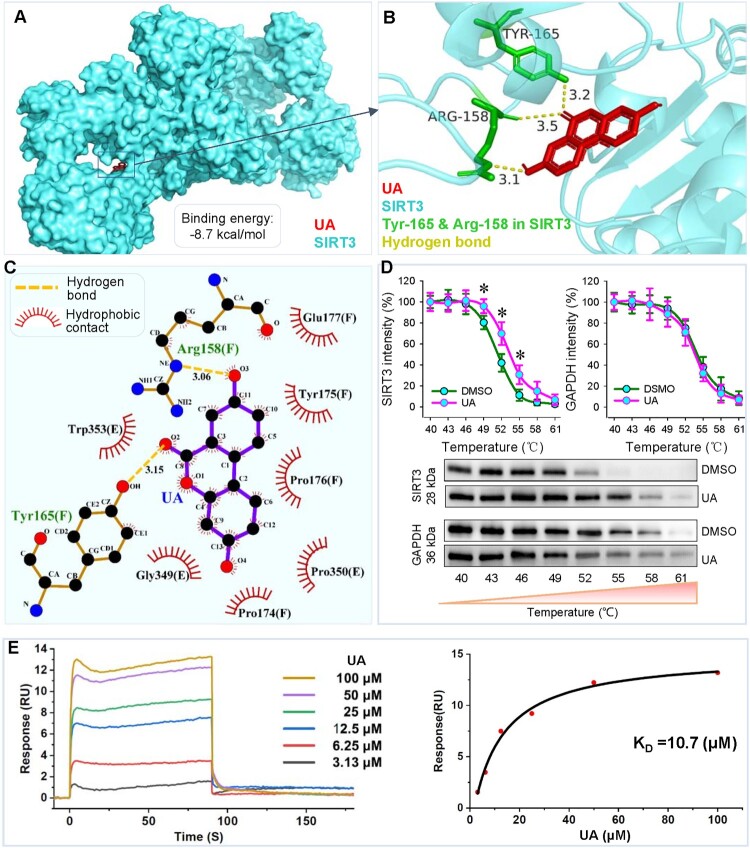
Molecular docking of UA and human SIRT3 protein. A, 3D structure showing the molecular docking of UA (red) and SIRT3 (cyan). B, substrate binding sites of UA with SIRT3. The 3D molecular docking model was made using AutoDock software and PyMOL software. Red, UA. Cyan, SIRT3. Green, amino acid residues (Try-165 and Arg-158) in SIRT3 protein, which bind to UA through hydrogen bonds. Yellow dashed line, hydrogen bond and the number near the line represents the bond distance. C, determinants of UA and SIRT3 for binding. The 2D molecular docking model was produced with LigPlot + software. The binding of UA to the amino acid residues (Try-165 and Arg-158) in the SIRT3 protein was realized through hydrogen bonds (Yellow dashed line). Hydrophobic contacts are represented with a red arc with spokes radiating. D, binding of UA to SIRT3 evaluated by the thermal stability of SIRT3 in VSMCs treated with 1% DMSO or UA (25 μM). E, Sensorgram of UA binding to SIRT3 protein examined with SPR. K_D_, dissociation constant. *P < 0.05 vs DMSO, *n* = 4 for D. Two-way ANOVA followed by Bonferroni post hoc test.

SOD2 is crucial for reducing mitoROS in hypertension, and SOD2 activity is regulated by sirtuin-3 (SIRT3) [37]. The SIRT3 mRNA is transcribed from nuclear DNA and translated in the cytoplasm, but the majority of SIRT3 proteins translocate to mitochondria [38]. SIRT3 depletion leads to the inactivation of vascular SOD2 due to hyperacetylation, and thus increases mitoROS level [37]. Molecular docking involves the docking of ligand and receptor in an active pocket, accompanied by a change in binding energy. The lower the binding energy is, the more stable the binding conformation. A docking binding energy less than 0 kcal/mol indicates that the receptor and ligand can bind spontaneously, while energy below −5 kcal/mol indicates good binding potential between the receptor and ligand [39]. Molecular docking 3D model showed that UA bound to SIRT3 with a strong affinity (binding energy: −8.7 kcal/mol) (Figure 4(A)). The 3D model produced by AutoDock software and PyMOL software showed that hydrogen bonds were formed between the UA and the Arg-158 and Tyr-165 residues of the SIRT3 protein (Figure 4(B)). The 2D model produced by LigPlot + software also showed the hydrogen bonds between the UA and the Arg-158 and Tyr-165 residues of SIRT3 protein. A strong hydrophobic effect was observed in the ligand binding domain of SIRT3 (Figure 4(C)). CETSA was performed to examine the thermal stability of SIRT3 treated with 1% DMSO and UA. UA significantly protected SIRT3 from temperature-dependent denaturation, supporting that UA directly interacts with SIRT3 (Figure 4(D)). Furthermore, the SPR assay showed the binding of UA to SIRT3 protein fixed on the surface of the sensor chip CM5 in a concentration-dependent manner, and the dissociation constant (K_D_) was 10.7 μM (Figure 4(E)). These results suggest that UA has a good affinity with the SIRT3 protein. SIRT3 may be involved in the effects of UA on SOD2 activity.

### Effects of SIRT3 inhibitor on the roles of UA

**Figure 5.**
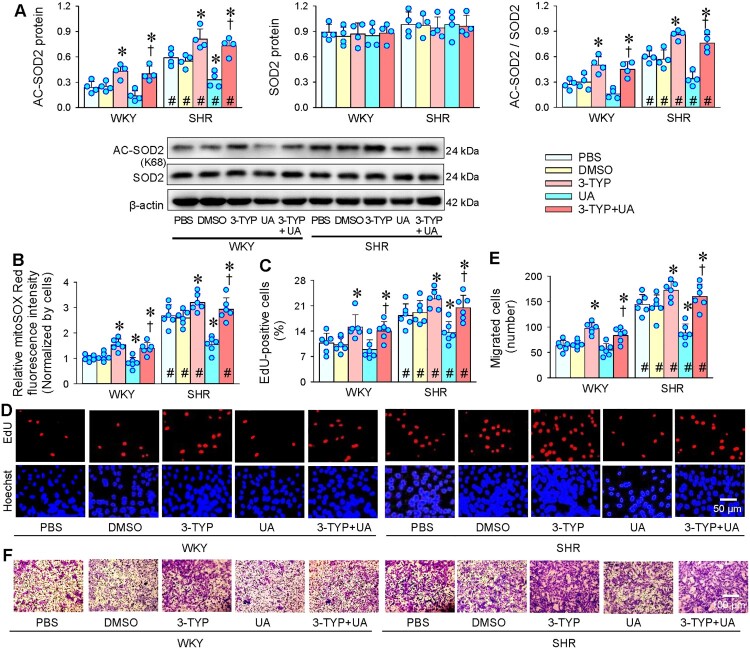
Effects of SIRT3 inhibitor 3-TYP on the UA-induced oxidative stress, proliferation and migration in VSMCs of WKY and SHR. 3-TYP (50 μM) was administered 1 h before UA (25 μM). A, acetylated SOD2 (AC-SOD2) and SOD2 protein expression. B, relative mitoSOX Red fluorescence intensity showing mitochondrial ROS levels. C & D, VSMC proliferation evaluated with EdU incorporation assay. E & F, VSMC migration evaluated with Boyden chamber assay. Values are mean ± SD. *P < 0.05 vs PBS or DMSO; †*P* < 0.05 vs UA alone; #*P* < 0.05 vs WKY. *n* = 4 for A; *n* = 6 for B, C, E. Two-way ANOVA followed by Bonferroni post hoc test.

SOD2 activity is mainly regulated by acetylation. The deacetylation increases SOD2 activity, and thus reduces mitoROS production [40]. 3-(1H-1,2,3-triazol-4-yl) pyridine (3-TYP) is a selective SIRT3 inhibitor, which reduces SIRT3 activity without significant impact on SIRT3 expression [41]. Acetylated SOD2 (AC-SOD2) level in VSMCs was higher in SHR than that in WKY, and UA reduced AC-SOD2 levels in both strains. 3-TYP increased AC-SOD2 levels and almost abolished the role of UA in reducing AC-SOD2 levels. There was no significant difference in the SOD2 protein expression between WKY and SHR, and UA had no significant effect on SOD2 expression (Figure 5(A)). Being consistent with the acetylation of SOD2, UA reduced mitoROS production in VSMCs of both WKY and SHR, and 3-TYP increased mitoROS production and almost abolished the role of UA in reducing mitoROS production (Figure 5(B)). UA inhibited VSMCs proliferation of SHR, which was prevented by 3-TYP (Figure 5(C,D)). Similarly, UA the inhibitory effect of UA on VSMCs migration of SHR was prevented by 3-TYP (Figure 5(E,F)). These findings indicate that the inhibitory effects of UA on VSMCs proliferation and migration were mediated by SIRT3 upregulation-dependent SOD2 deacetylation and subsequent decline in mitoROS level.

### Effects of UA on SIRT3 expression and localization

**Figure 6.**
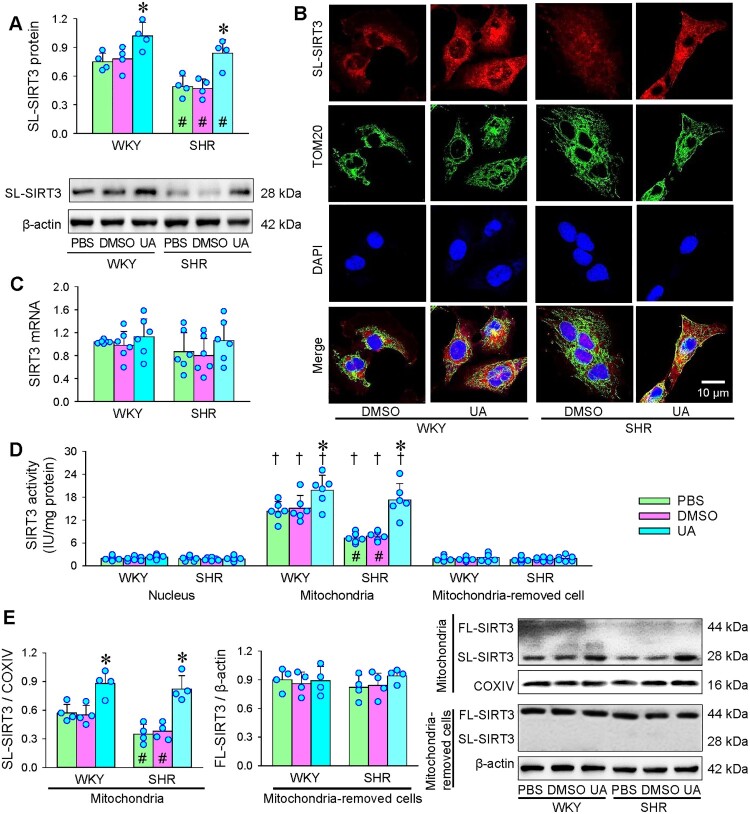
Effects of UA on SIRT3 expression, localization and activation in VSMCs of WKY and SHR. Measurements were made 24 h after administration of PBS, DMSO or UA (25 μM). A, SL-SIRT3 protein in VSMCs. B, immunofluorescence analysis for SL-SIRT3 in VSMCs. Red, SL-SIRT3; Green, TOM20 (a marker of mitochondria); Blue, DAPI (a marker of nucleus). C, SIRT3 mRNA levels in VSMCs. D, SIRT3 activity in nucleus, mitochondria and mitochondria-removed cells. E, FL-SIRT3 (44 kDa) and SL-SIRT3 (28 kDa) expressions in mitochondria, and mitochondria-removed cells. Values are mean ± SD. *P < 0.05 vs PBS or DMSO; †*P* < 0.05 vs Nucleus or Mitochondrial-removed cell; #*P* < 0.05 vs WKY. *n* = 4 for A. B, E. *n* = 6 for C, D. Two-way ANOVA followed by Bonferroni post hoc test.

SIRT3 is a member of the sirtuin family. Full-length SIRT3 (FL-SIRT3, 44 kDa) is an inert protein, which is activated in mitochondria following deletion of 142 amino acids of the N-terminal segment by mitochondrial processing peptidase to form short-length SIRT3 (SL-SIRT3, 28 kDa) [42]. SL-SIRT3 is the active form of SIRT3, and mainly locates in mitochondria [38]. SL-SIRT3 level in the whole-cell lysates of VSMCs was reduced in SHR compared with WKY, and UA increased SL-SIRT3 levels in both strains (Figure 6(A)). Immunofluorescence analysis showed that SL-SIRT3 is mainly located in mitochondria, and UA increased mitochondrial SL-SIRT3 expression in both WKY and SHR (Figure 6(B)). However, UA had no significant effect on SIRT3 mRNA levels in VSMCs of both WKY and SHR (Figure 6(C)). SIRT3 activity was very low in the nucleus and mitochondria-removed cells of VSMCs, but very high in the mitochondria. The SIRT3 activity in mitochondria was lower in the VSMCs of SHR than in those of WKY. UA increased SIRT3 activity in the mitochondria of both WKY and SHR. However, UA had no significant effects on SIRT3 activity in the nucleus and the mitochondria-removed cells (Figure 6(D)). To further determine the subcellular localization of two types of SIRT3 in the cells, a kind of SIRT3 antibodies showing both FL-SIRT3 and SL-SIRT3 was used for Western blotting analysis. SL-SIRT3 was rich in the mitochondria lysates of VSMCs, but FL-SIRT3 was trace. Conversely, FL-SIRT3 was abundant in the lysates of the mitochondria-removed cells, but SL-SIRT3 was trace. The SL-SIRT3 level in mitochondria was downregulated in SHR compared with that in WKY, and UA increased the SL-SIRT3 levels in both strains. However, UA had no significant effects on the FL-SIRT3 expressions (Figure 6(E)). These results suggest that UA increased SIRT3 activity mainly by promoting the conversion of FL-SIRT3 to SL-SIRT3 in mitochondria, rather than by promoting the expression of SIRT3.

### Relationship between mitophagy and reduced mitoROS level induced by UA

**Figure 7.**
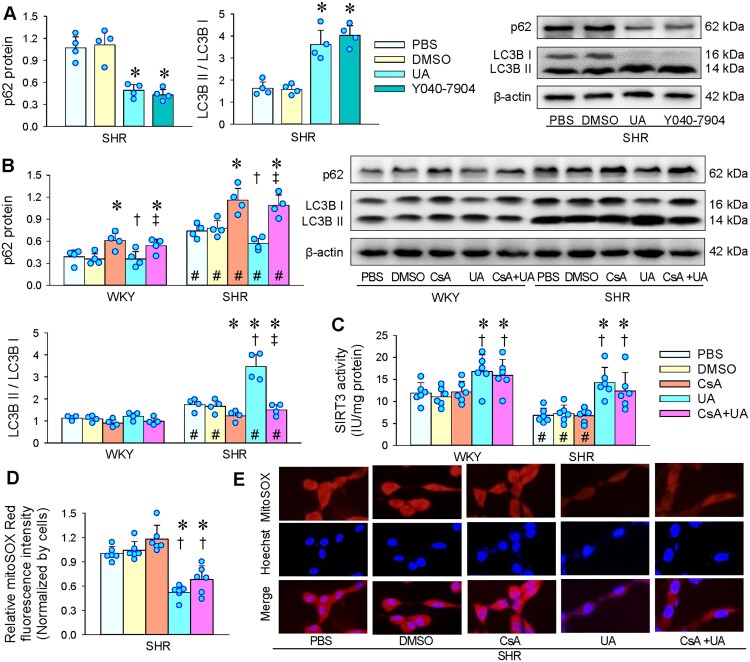
Relationship between mitophagy and reduced mitoROS level induced by UA in VSMCs. Measurements were made 24 h after administration of PBS, DMSO, UA (25 μM), Y040-7904 (20 μM) or Cyclosporin A (CsA, 2 μM). A, Comparison of the effects of UA and specific mitophagy inducer Y040-7904 on mitophagy marker p62 and LC3B II / LC3B I in VSMCs of SHR. B, Effects of inhibition of PINK1/Parkin signaling-mediated mitophagy with CsA on UA-induced mitophagy in VSMCs of WKY and SHR. C, Effects of CsA on UA-induced SIRT3 activation in VSMCs of WKY and SHR. D, Effects of CsA on the role of UA in reducing mitoROS levels in VSMCs of SHR. E, representative images showing the mitoROS levels (red) in VSMCs of SHR. Nuclei were stained with Hoechst (blue). Values are mean ± SD. *P < 0.05 vs PBS or DMSO; †*P* < 0.05 vs CsA; ‡*P* < 0.05 vs UA; #*P* < 0.05 vs WKY. *n* = 4 for A & B; *n* = 6 for C & D. One-way ANOVA (A & D) and two-way ANOVA (B & C) followed by Bonferroni post hoc test.

UA is known to be a mitophagy activator, and oral consumption of UA shows a molecular signature of improved mitochondrial and cellular health in humans [43,44]. PINK1/Parkin-mediated mitophagy improves palmitic acid-induced apoptosis by reducing mitoROS in podocytes [45]. It is interesting to know whether UA-induced mitophagy is involved in the reduced mitoROS level. UA had a similar effect to specific a mitophagy inducer Y040-7904 in reducing p62 protein expressions and increasing the ratio of LC3B II to LC3B I (Figure 7(A)), which was almost prevented by the inhibition of PINK1/Parkin signaling-mediated mitophagy with Cyclosporin A (CsA) (Figure 7(B)). CsA had no significant effects on the UA-induced SIRT3 activation (Figure 7(C)) and mitoROS reduction (Figure 7(D,E)). These results indicate that the role of UA in promoting SIRT3 activation and mitoROS reduction is independent of PINK1/Parkin signaling-mediated mitophagy.

### Effects of UA on blood pressure and vascular remodeling in SHR

**Figure 8.**
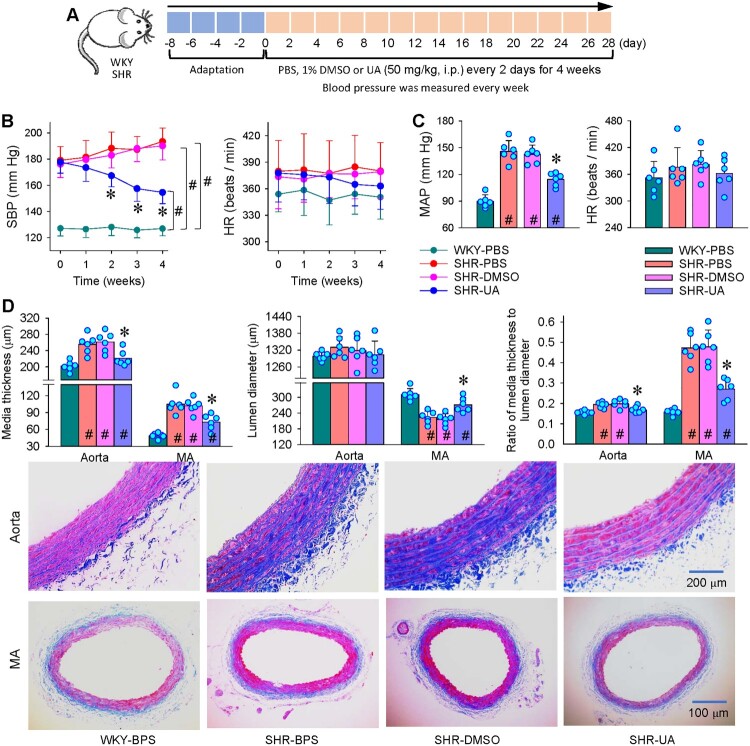
Effects of repeated intraperitoneal injection of UA on blood pressure and vascular remolding in SHR. A, schematic diagram showing the protocol of UA intraperitoneal injection (50 mg/kg, every 2 days for 4 weeks). B, systolic blood pressure (SBP) of tail artery and heart rate (HR) measured every week under the waking state. C, mean arterial pressure (MAP) of the common carotid artery and heart rate (HR) measured under anesthesia. D, Masson’s stain analyses showing media thickness, lumen diameter and their ratio of aorta and mesenteric artery (MA). Values are mean ± SD. *P < 0.05 vs SHR-PBS or SHR-DMSO; #*P* < 0.05 vs WKY-PBS. *n* = 6. One-way ANOVA (C) or Two-way ANOVA (B, D) followed by Bonferroni post hoc test.

Repeated intraperitoneal injection of UA (50 mg/kg) was performed in SHR every 2 days for 4 weeks to determine whether long-term administration of UA might attenuate blood pressure and vascular remodeling in SHR (Figure 8(A)). UA reduced blood pressure in SHR either in the waking state or under anesthesia, but had no significant effects on HR (Figure 8(B and C)). The significant antihypertensive effects occurred 2 weeks after the administration of UA (Figure 8(B)). UA attenuated the PCNA upregulation in the MA of SHR (Figure S2). Masson’s staining analyses showed that UA reduced the media thickness and the ratio of media thickness to lumen diameter in the aorta and MA of SHR. Furthermore, UA rectified the reduced lumen diameter in MA of SHR (Figure 8(D)). These results indicate the beneficial roles of UA in the antihypertension and the attenuation of vascular remodeling in SHR.

### Effects of UA on vascular oxidative stress in SHR

**Figure 9.**
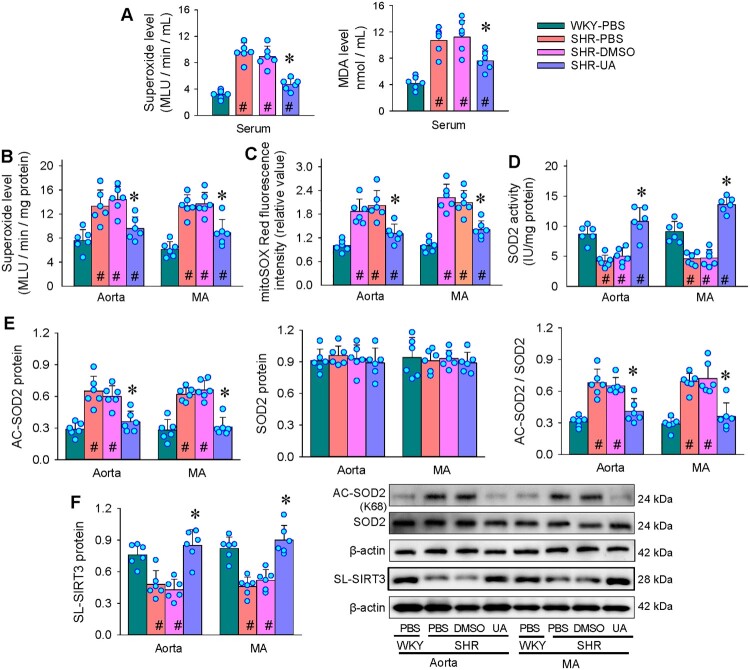
Effects of repeated intraperitoneal injection of UA on vascular oxidative stress in SHR. UA (50 mg/kg) was injected every 2 days for 4 weeks. A, superoxide level and MDA level in serum. B, superoxide level of aorta and mesenteric artery (MA). C, mitochondrial ROS level of aorta and MA. D, SOD2 activity. E, acetylated SOD2 and SOD2 protein expression in aorta and MA. F, SL-SIRT3 protein expression in aorta and MA. Values are mean ± SD. *P < 0.05 vs SHR-PBS or SHR-DMSO; #*P* < 0.05 vs WKY-PBS. *n* = 6. One-way ANOVA (C) or Two-way ANOVA (B, D) followed by Bonferroni post hoc test.

Repeated intraperitoneal injection of UA was performed in SHR (50 mg/kg, every 2 days for 4 weeks). Serum superoxide and malondialdehyde (MDA) levels were increased in SHR compared with WKY, which were attenuated by UA (Figure 9(A)). The increased superoxide production and mitoROS level in aorta and MA of SHR were almost prevented by the repeated injection of UA (Figure 9(B and C)). SOD2 activity was reduced in both arteries of SHR, which was reversed by UA (Figure 9(D)). Correspondingly, the increased SOD2 deacetylation levels in aorta and MA of SHR were prevented by UA (Figure 9(E)). SL-SIRT3 protein levels in the aorta and MA were reduced in SHR, which was prevented by the UA (Figure 9(F)). These results suggest that UA attenuates vascular remodeling of SHR via SIRT3-SOD2 signaling pathway.

## Discussion

**Figure 10.**
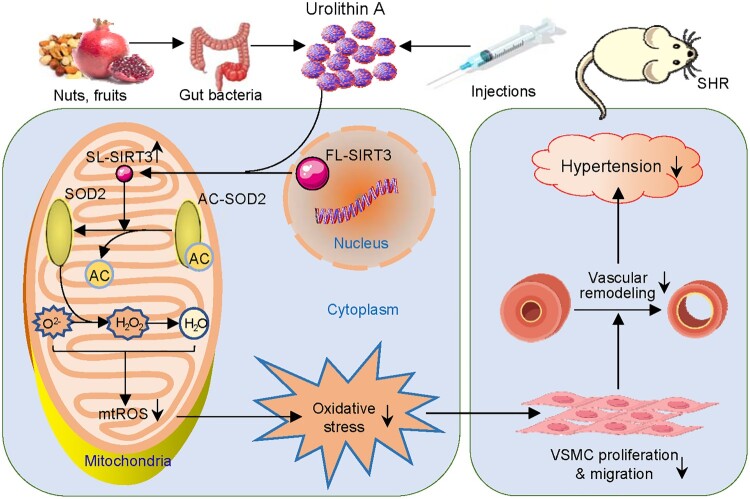
Diagrammatic sketch showing the roles of urolithin A (UA) in attenuating oxidative stress, VSMC proliferation and migration, and vascular remodeling in SHR.

UA is a natural polyphenolic compound produced by gut bacteria with anti-tumor, anti-inflammatory, antioxidant, and anti-aging properties [5]. Vascular remodeling contributes to the development of hypertension and cardiovascular events [6], while VSMCs proliferation and migration are important pathological processes in the vascular remodeling [10,35]. The primary novel findings are that UA attenuates VSMCs proliferation and migration of SHR by increasing mitochondrial SL-SIRT3 level, which leads to SOD2 deacetylation and a decrease in mitochondrial ROS level (Figure 10). Long-term administration of UA in SHR attenuates oxidative stress, vascular remodeling and hypertension.

Various studies have shown that UA has anticancer effects against hepatic, gastric colorectal, prostate and breast cancers through multifaceted mechanisms including the modulation of autophagy and apoptosis, inhibition of proliferation, migration and inflammation, and activation of tumor suppressors [46–48]. We found that UA attenuated the proliferation, migration and oxidative stress in the VSMCs of SHR. UA only mildly reduced the increased NOX activity and NOX1 expression in VSMCs of SHR rather than those of WKY, and UA had no significant effects on NOX2 and NOX4 expressions in both WKY and SHR. It is noted that UA reduced mitoROS in the VSMCs of both WKY and SHR, but had no significant effect on NOX activity and NOX1 expression in the VSMCs of WKY. These results suggest that the inhibitory effect of UA on oxidative stress in the VSMCs of SHR may be mainly attributed to the reduction of mitoROS, and NOX1 may only play a small role in the reduction of cellular ROS.

Mitochondria are a major site for redox homeostasis between ROS production and scavenging [49]. SOD2 is the principal antioxidant enzyme for scavenging ROS in mitochondria [50]. UA had no significant effects on SOD2 protein expression. However, UA increased SOD2 activity but reduced mitoROS level, which was almost prevented by the knockdown of SOD2 in the VSMCs of both WKY and SHR. Importantly, SOD2 knockdown not only promoted VSMCs proliferation and migration, but also prevented the roles of UA in attenuating VSMCs proliferation and migration in SHR. These findings indicate that the modulation of SOD2 activity is the main target of UA, and SOD2 activation is responsible for the roles of UA in inhibiting mitoROS production, proliferation and migration in VSMCs of SHR.

SOD2 activity is inhibited through acetylation, which is mainly modulated by SL-SIRT3 in mitochondria [37]. SIRT3 is activated by the conversion of FL-SIRT3 to SL-SIRT3 upon entering mitochondria [42]. Molecular docking analysis suggests that UA has a strong affinity with the SIRT3 protein. UA increased SIRT3 activity and mitochondrial SL-SIRT3 level, but had no significant effects on SIRT3 mRNA levels in the VSMCs of both WKY and SHR. It had no significant effects on SOD2 protein expression, but inhibited SOD2 acetylation, SOD2 activity and mitoROS production, which were almost abolished by SIRT3 inhibitor 3-TYP. More importantly, the roles of UA in inhibiting VSMCs proliferation and migration in SHR were prevented by the inhibition of SIRT3 activity. These results indicate that UA increases SL-SIRT3 formation in mitochondria, which leads to deacetylation of SOD2, decline of mitoROS level, and inhibition of VSMCs proliferation and migration in SHR.

Intraperitoneal injection of UA was performed every 2 days for 4 weeks to examine the long-term effects of UA in SHR. Long-term administration of UA attenuated hypertension and vascular remodeling of SHR. It increased vascular SL-SIRT3 level, promoted SOD2 acetylation, increased SOD2 activity, and reduced vascular ROS and mitoROS levels in SHR. These results indicate that UA plays beneficial roles in attenuating vascular remodeling and hypertension in SHR, and further support the in vitro findings that SIRT3-SOD2-mitoROS signaling contributes to the beneficial effects of UA. It is known that hypertension promotes vascular remodeling, while vascular remodeling aggravates hypertension [8,51]. It is noted that UA did not show a significant antihypertension effect in the first week. We propose that the antihypertension effect of UA might be primarily secondary to its role in attenuating the vascular remodeling effect. The roles of UA in inhibiting VSMCs proliferation, migration and oxidative stress at least partially contribute to its beneficial effect on attenuating vascular remodeling in SHR. A limitation in the present study is that the roles of UA were examined in animal models, and the findings are not necessarily applicable to human hypertension, which needs further investigation.

In summary, UA attenuates VSMCs proliferation and migration of SHR via reducing mitochondrial ROS level, which was mediated by mitochondrial SL-SIRT3 formation and following deacetylation of SOD2. Long-term administration of UA attenuates vascular remodeling, hypertension and oxidative stress in hypertension.

## Supplementary Material

Online supplmentary data.docx

## Data Availability

The data supporting the findings of this study are available from the corresponding author on reasonable request.
