# Supplementary material for: Urolithin A alleviates vascular remodeling through mitochondrial SIRT3-mediated SOD2 deacetylation and antioxidation in hypertensive rats
Source: Redox Rep. 2026 Feb 6;31(1):2622255. doi: 10.1080/13510002.2026.2622255 (PMC12885030; doi:10.1080/13510002.2026.2622255)
Supplement: Online supplmentary data.docx [file YRER_A_2622255_SM6694.docx]

**Online supplementary data**

**
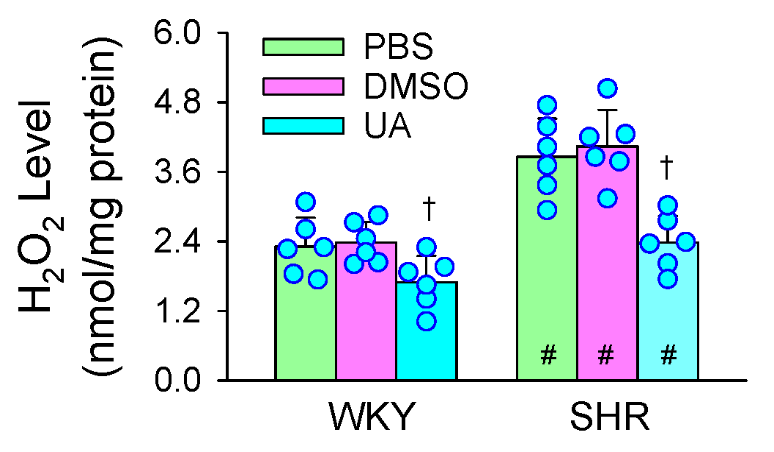
**

**Figure S1 Effects of UA on H_2_O_2_ level in VSMCs of WKY and SHR.** Values are mean±SD. †P<0.05 vs PBS or DMSO; #P<0.05 vs WKY. n=6. Two-way ANOVA followed by Bonferroni post hoc test.

**
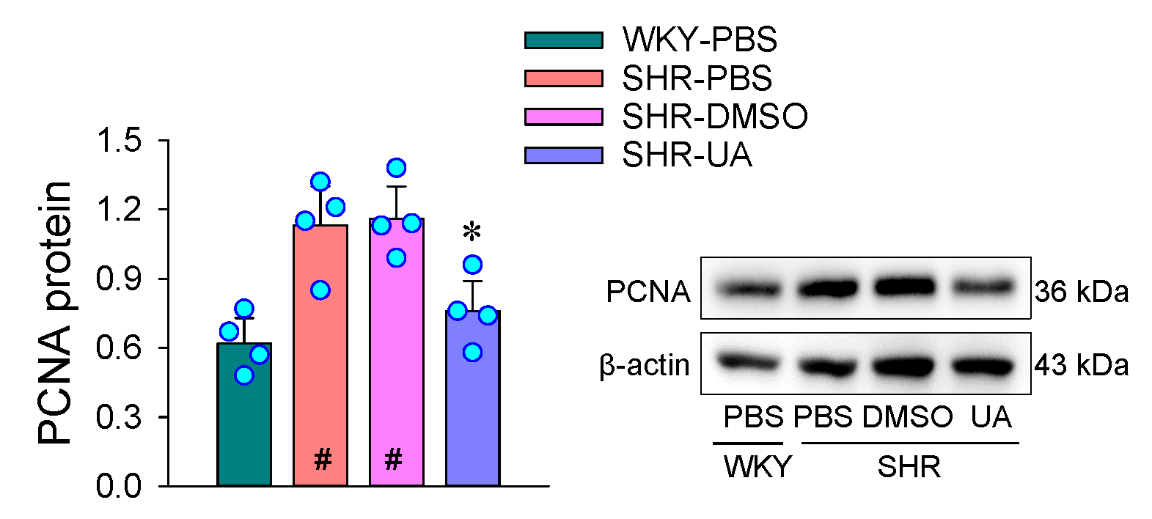
**

**Figure S2** Effects of repeated injection of UA on PCNA protein expressions in mesenteric artery of SHR. UA (50 mg/kg) was injected intraperitoneally every 2 days for 4 weeks. WKY treated with PBS (WKY-PBS), SHR treated with PBS (SHR-PBS), and SHR treated with 1% DMSO (SHR-DMSO) were used as controls of SHR treated with UA (SHR-UA). Values are mean±SD. **P*<0.05 vs SHR-PBS or SHR-DMSO; #*P*<0.05 vs WKY-PBS. n=4. One-way ANOVA followed by Bonferroni post hoc test.
